# Can radiomics outperform radiologists in predicting Ewing sarcoma response to neoadjuvant chemotherapy from longitudinal MRI?

**DOI:** 10.1186/s12885-026-17332-0

**Published:** 2026-11-12

**Authors:** Óscar Llorián-Salvador, Daniel Rusche, Hans R. Dürr, Nicole Martin, Johanna Luitjens, Alexander Klein, Felix Gassert, Florian Gassert, Stefan Weissinger, Miguel A. Andrade-Navarro, Carolin Knebel, Stephanie E. Combs, Burkhard Rost, Alexandra S. Gersing, Jan C. Peeken

**Affiliations:** 1 Department of Radiation Oncology, School of Medicine and Health, TUM University Hospital, Technical University of Munich, Ismaninger Straße 22, 81675 Munich, Germany; 2 Department of Informatics, Bioinformatics and Computational Biology—i12, Technische Universität München, Boltzmannstr. 3, 85748 Munich, Germany; 3 Institute of Organismic and Molecular Evolution, Johannes Gutenberg University Mainz, Hanns-Dieter-Hüsch- Weg 15, 55128 Mainz, Germany; 4 Musculoskeletal Oncology, Department of Orthopaedic and Trauma Surgery, Physical Medicine and Rehabilitation, University Hospital Munich (LMU), Marchioninistrasse 15, 81377 Munich, Germany; 5 Department of Radiology and Biomedical Imaging, University of California, San Francisco, CA USA; 6 Department of Diagnostic and Interventional Neuroradiology, University Hospital Munich (LMU), Marchioninistrasse 15, 81377 Munich, Germany; 7 Orthopaedic Oncology, Department of Orthopaedics and Trauma Surgery, LMU University Hospital, Marchioninistrasse 15, 81377 Munich, Germany; 8 Department of Diagnostic and Interventional Radiology, School of Medicine and Klinikum Rechts der Isar, Technical University of Munich, Munich, Germany; 9 Department of Orthopedics and Sports Orthopedics, Klinikum Rechts der Isar, Technische Universität München, Ismaninger Straße 22, 81675 Munich, Germany; 10 German Cancer Consortium (DKTK), Partner Site Munich and German Cancer Research Center (DKFZ), Munich, Germany; 11 German Research Center for Environmental Health, Helmholtz Zentrum München (HMGU) GmbH, Institute of Radiation Medicine (IRM), Munich, Germany

**Keywords:** Radiology, Radiomics, Machine learning, Ewing sarcoma, MRI, Neoadjuvant therapy, Histological response

## Abstract

**Objective:**

This retrospective, multicenter study evaluated whether MRI-based radiomics can predict the histological response to neoadjuvant chemotherapy in Ewing sarcoma patients and compared their performance to that of radiological findings. Definitive histological assessment is only possible during surgery, potentially delaying therapy changes and increasing metastatic risk; therefore, a positive outcome would enable early adaptations.

**Methods:**

Radiomics features were extracted from pre- and post-neoadjuvant (after first chemotherapy cycle) chemotherapy scans of patients treated from 2004 to 2020. Four machine learning models were trained using nested cross-validation to predict histological response, and their performance was compared to models trained on semantic features derived from expert radiological readings. Diagnostic performance was measured by the area under the receiver operating characteristic curve (AUROC).

**Results:**

96 patients (mean age, 19.9 ± 11.7 years; 41 women) with Ewing sarcoma were evaluated. Radiomics features related to low gray level intensity changes in T1 fat-saturated contrast-enhanced sequences showed the highest responder identification performance (AUROC, 0.62; 95% CI: 0.59, 0.65), surpassing the best radiological model, which relied on the relative change in maximum diameter of extratumoral soft tissue components (AUROC, 0.58; 95% CI: 0.56, 0.60). Radiomics models relying on pre-treatment T2-weighted data performed similarly (AUROC, 0.61; 95% CI: 0.58, 0.64), outperforming the clinical baseline (AUROC, 0.53; 95% CI: 0.51, 0.55).

**Conclusion:**

MRI-based radiomics moderately identified responders to neoadjuvant chemotherapy, suggesting a role for earlier, tailored therapeutic strategies in Ewing sarcoma. Larger studies to validate these findings within a multimodal framework are warranted.

**Clinical relevance statement:**

Radiomics extracted from MRI scans may aid with non-size tissue signals in early prediction of neoadjuvant chemotherapy response in Ewing sarcoma patients, potentially guiding personalized treatment adaptations, reducing delays for non-responders, and improving patient outcomes. These exploratory findings are complementary to standard size-based assessment from clinical routine, and warrant larger, prospective trials to be established.

**Supplementary Information:**

The online version contains supplementary material available at https://doi.org/10.1186/s12885-026-17332-0.

## Key points

Current diagnostic methods cannot assess Ewing sarcoma chemotherapy response until surgery, potentially delaying crucial treatment adjustments and increasing metastatic risk.

MRI-based radiomics features captured histological response to chemotherapy better than semantic features extracted from radiological readings assessments, especially from pre-neoadjuvant therapy T2-weighted images and from relative changes in T1-weighted contrast-enhanced images. Performance was modest and mainly complements the established size-based assessment for overall treatment response.

Radiomics can enable early identification of chemotherapy non-responders suffering from Ewing sarcomas, potentially leading to timely personalized treatment modifications. This can improve patient outcomes by reducing ineffective therapy duration and minimizing the risk of disease progression.

**Supplementary Information:**

The online version contains supplementary material available at https://doi.org/10.1186/s12885-026-17332-0.

## Introduction

Medical imaging plays a critical role in oncology, providing essential tools for the diagnosis, staging, and monitoring of various cancers. Modalities such as computed tomography (CT), magnetic resonance imaging (MRI) and positron emission tomography (PET) offer structural, functional and anatomical insights that guide clinical management [1]. Beyond conventional radiological evaluation, the evolving field of radiomics and machine learning has significantly expanded the utility of medical imaging. Artificial intelligence has demonstrated high accuracy in automating complex imaging tasks, such as deep reinforcement learning for organ localization in CT scans [2] and neural networks for automated lesion segmentation in CTs and MRIs [3, 4]. Furthermore, radiomics has repeatedly proven to be a robust tool for predicting treatment outcomes, particularly in assessing responses to neoadjuvant chemotherapy across diverse oncological applications [5].

While these advanced predictive techniques show broad applicability across adult oncology, they also hold significant potential for specific pediatric and adolescent malignancies. Ewing sarcoma is the second most frequent bone sarcoma in children and adolescents after osteosarcoma [6]. There are several treatment strategies, such as radiation therapy [7], though the most common treatment procedure consists of neoadjuvant chemotherapy, surgical resection and adjuvant chemotherapy. To monitor the effect of neoadjuvant therapies, MRI scans are performed before neoadjuvant therapy as a baseline examination, and a few weeks after the first chemotherapy cycles to monitor the response to treatment. Other monitoring modalities include the use of PET and PET/CT [8].

Unfortunately, a definitive histological reference for the response to chemotherapy can only be obtained during surgical resection. As a result, non-responders spend valuable time during the extended neoadjuvant treatment before receiving resection, even risking metastatic spread. Therefore, a prediction of the response to the first neoadjuvant treatment based on imaging characteristics would be clinically relevant and can potentially improve the clinical outcome.

Recent studies showed that MRIs can be used to evaluate the response to neoadjuvant chemotherapy and that machine learning methods can reliably aid in predicting therapy outcomes in patients with sarcomas [9–11]. However, these analyses were either conducted on relatively small datasets, which may have limited the generalizability of such findings, or studied exclusively MRI-based radiomics features.

Ewing sarcoma response assessment in practice is primarily size-driven (e.g., via RECIST) and guides therapy and its adaptation; histologic viability after induction remains an important prognostic factor in contemporary protocols (for instance in the EURO EWING 2012 trial [12]), where poor response (≥ 10% viable cells) and volume thresholds (≥ 200 mL) or < 50% radiologic regression stratify risk and treatment intensity. Our study treats histologic viability as a biologic correlate, not a stand-alone definition of overall response.

The aim of this study is to assess the predictive power of radiomics features obtained from MRIs before and after the first cycle of chemotherapy to predict the histological response of patients with Ewing sarcoma. In addition, we aim to compare the results with models based on radiological findings assessed by experts. Finally, we analyzed the predictive value of the most important radiomics features, as well as those extracted from radiological readings.

## Materials and methods

The local institutional review board (IRB) approved this retrospective multi-center study (ethics committee 21–0282). The study was performed in accordance with our institutional ethic guidelines and the 1964 Declaration of Helsinki and its later amendments. Written and informed consent was waived for this retrospective anonymized analysis.

### Dataset collection and preparation

Patient databases from Ludwig-Maximilians-University (LMU) hospital and Technical University of Munich hospital rechts der Isar (TUM) were retrospectively searched for bone tumor patients treated with VIDA scheme neoadjuvant chemotherapy between 2004 and 2020. Patients treated with neoadjuvant chemoradiotherapy or radiotherapy were excluded, as well as patients with histological diagnosis other than Ewing sarcoma or insufficient imaging. In total, *n* = 96 patients met the clinical criteria (see patient workflow in Figure S1). Histological response, as a complementary tissue-signal information to size/volume for overall treatment response, was divided into two groups depending on whether there was a change in the percentage of vital tumor cells after treatment greater than or equal to 10% (grade 3 or higher from the Salzer-Kuntschnik score [13]) or not (grade lower than 3).

### MR Imaging protocol and image segmentation

**Fig. 1:**
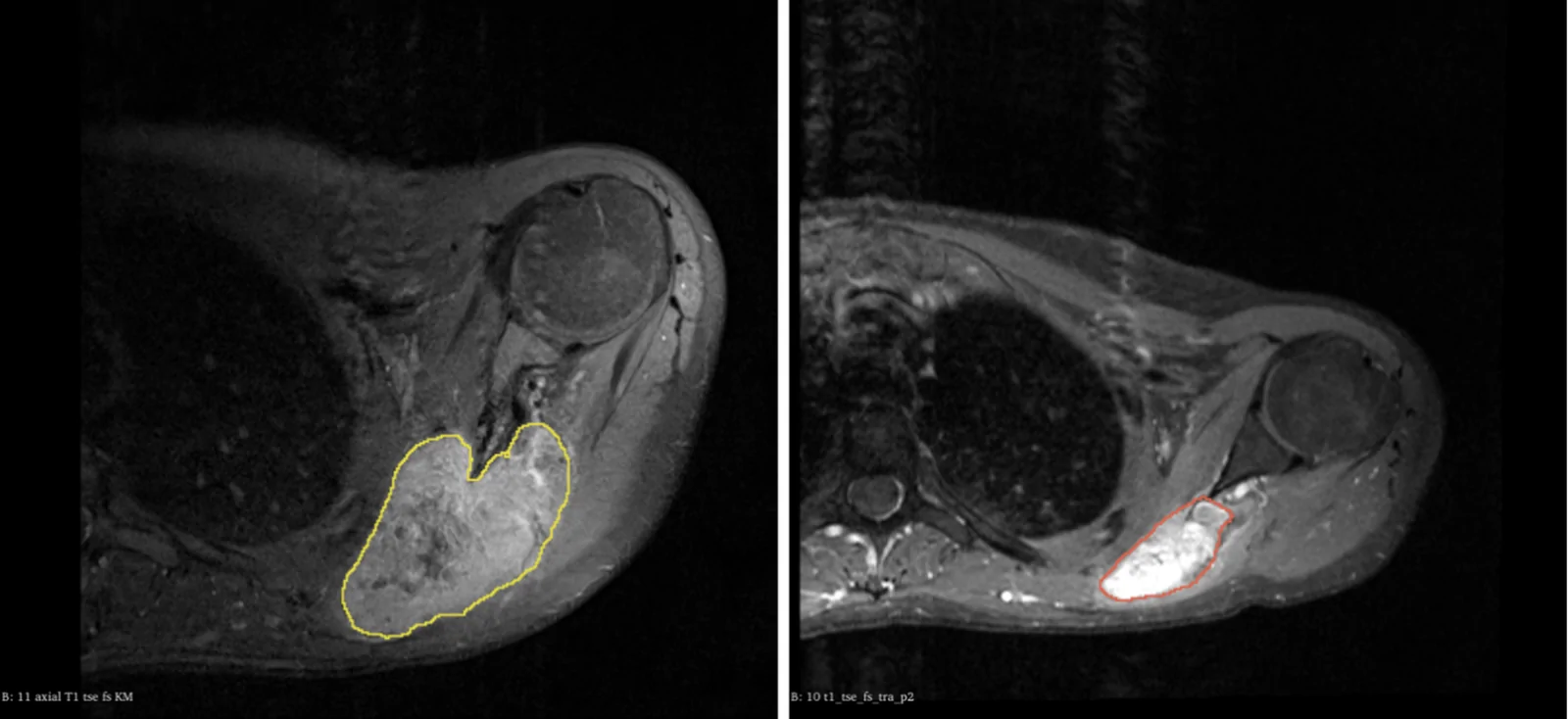
Example of the tumor volume segmentations in a representative patient from the present study with Ewing sarcoma located at the scapula at baseline (left) and after the first cycle of chemotherapy (post-ct; right)

Imaging data consisted of pre-therapeutic baseline and post-ct MRI using 1.0, 1.5 and 3.0 Tesla scanners. We considered three sequences: T1-weighted imaging without contrast agent (T1w), T1-weighted fat-saturated imaging with the administration of contrast agent (T1fsgd) and T2-weighted imaging (T2w). More details regarding the MR imaging acquisition parameters can be seen in Table S1. The available Digital Imaging and Communications in Medicine (DICOM) files were exported and the volumes of interest (VOI) were segmented by two radiology residents (J.L. and F.G., both 3 years of experience in musculoskeletal imaging) with the support of a research assistant (S. W.), and using the software 3D Slicer (version 4.11) [14] as illustrated in Fig 1. Segmentation label maps were extracted as neuroimaging informatics technology initiative (NIfTI) files. For 20 cases segmentation was performed twice with more than 4 weeks between the readings by J.L. and F.G. for later computation of inter-rater variability.

### Morphological analysis

The following morphological radiological features were extracted at the baseline and after the first chemotherapy cycle (post-ct) time points: Bone, Location within the bone, Location of affected bone, Sharp border, Intramedullary fat present, Diameter maximum, Diameter minimum, Growth pattern, T1 signal without GD, T1 + GD enhancement, T2 signal, Lobulation, Periostal reaction, Expansion, Edema in bone, Edema soft tissue, Extraosseous soft tissue component, Extratumoral soft tissue components (maximum diameter), Cortical involvement and Necrosis.

### Feature sets extraction

Feature extraction and the implementation of the machine learning pipeline in this project were based on Python3 (version 3.8.10) [15]. We extracted a total of 104 features for each sequence at baseline and post-ct respectively, following IBSI recommendations [16] (SimpleITK, version 2.2.0 [17]; Pyradiomics, version 3.0.1 [18]). We included first order, shape and texture features (Table S2). A clinical set was established to serve as a baseline for benchmarking the performance of all trained models in this study. This baseline clinical set contained the following features: sex, age, and presence of metastasis. In addition to the Response Evaluation Criteria in Solid Tumors (RECIST) [19] derived from T2w, the mesh volume of the tumor was evaluated as a predictor. To account for inter-rater variability, features with an intraclass correlation (3,1) < 0.8 were excluded (ICC; Pingouin, version 0.5.3) [20].

All feature sets were transformed to the standard normal distribution using min-max normalization (Scikit-learn library version 1.0.2) [21]. Feature normalization was performed before splitting the data for training following batch harmonization requirements. Using MRI scanner names extracted from DICOM files with Pydicom (version 2.3.0) [22] we performed harmonization of the combined feature sets of a sequence at baseline and post-ct using Combatbatch harmonization (version 0.2.1) [23] to compensate for variations introduced by different brands of MRI scanners. Distributions of brands of MRI scanners and their magnetic field strengths are shown in Tables S5 and S6. Absolute delta ($\:\mathrm{post-ct}\:-\mathrm{baseline}$) and relative delta ($\:\left(\mathrm{post-ct}-baseline\right)\:/\mathrm{\:baseline}$) time points for the feature sets were also calculated to explore the predictive quality of feature evolution.

Three trained radiologists performed two sets of readings for each MR imaging scan (Table 1; J.L. performed one reading, and F.G. and F.G. performed the second reading) which amounted to 19 radiological semantic features from each rater. The resulting continuous semantic features (e.g., measurements) susceptible to reading variations were excluded using the ICC 3,1 statistic with a threshold of 0.8 [24]. Discrete semantic features were tested for inter-rater variability using the Cohen’s Kappa test with a threshold of 0.4 [25]. Categorical features, prior to this test, were transformed using binary encoding. The remaining semantic features that passed the corresponding tests were used to train a radiological model that followed the same configuration as the best performing radiomics model. Patients with missing data were excluded from the respective feature set.

### Machine Learning modeling and statistical analysis

Before splitting and resampling the data, outlier detection was conducted to filter out extreme values that could influence the data distribution (Scikit-learn library version 1.0.2).

**Fig. 2:**
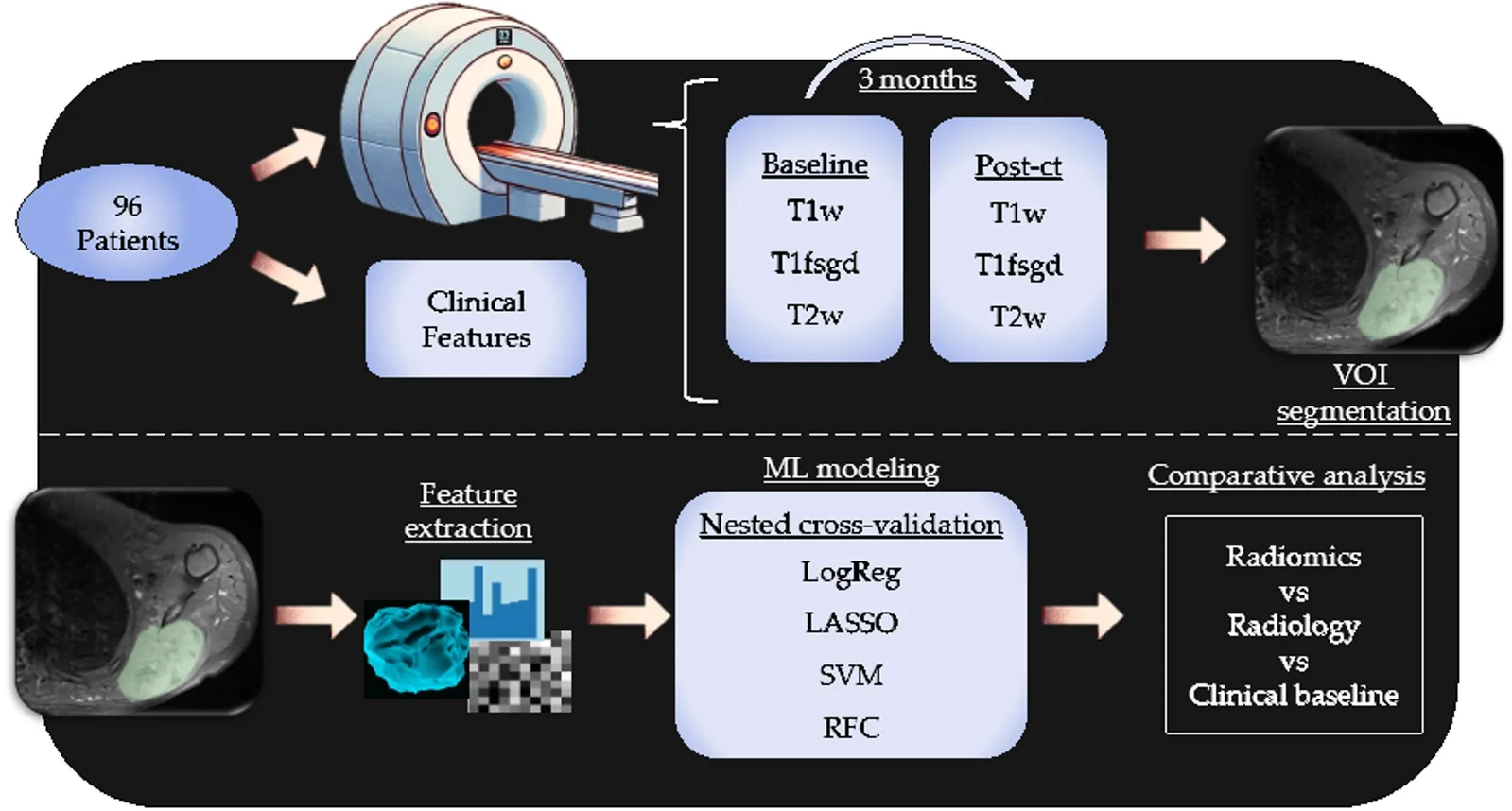
Machine learning modeling pipeline, showing the feature sets analyzed in this study. For 96 patients, clinical features and MRI scans were collected at the baseline and after the first cycle of chemotherapy (post-ct). Both scans include T1w, T1fsgd, and T2w sequences. Volumes of interest were segmented, and radiomics features were extracted from them. Clinical and radiomics features were used with nested cross-validation using logistic regression (LogReg), least absolute shrinkage and selection operator (LASSO), support vector machine (SVM) and random forest (RFC).

The radiomics and clinical features sets were evaluated for their predictive value for positive evolution of histological response using four different ML models (Fig. 2): logistic regression (LogReg), least absolute shrinkage and selection operator (LASSO), support vector machine (SVM) and random forest (RFC). The data were split and resampled using a 3-fold repeated nested cross-validation. In the inner fold, feature selection and imbalance correction were conducted to respectively reduce redundancy of the data and offset the imbalance of classes in the histological response. A total of 50 iterations of the training process were conducted for each model and set of features, resulting in a total of 150 combinations for each case.

Two different feature selection techniques were evaluated: a two-step Spearman’s rank correlation coefficient, to first exclude redundant features and then exclude low relevance features; and minimum redundancy-maximum relevance (MRMR). The number of features to be selected was approximated based on the variance density from the principal component analysis (PCA).

To offset the various degrees of class imbalance of the training set (ranging from 1.42:1 to 2.06:1), a combination of synthetic minority oversampling technique (SMOTE; imbalanced-learn library version 0.8.0) [26] and random undersampling of the majority class, or class weights, were used. Balanced accuracy (BA) was the metric used to optimize the hyperparameters of the models. SMOTE was explicitly chosen over boundary-focused methods to prevent the over-amplification of noise and outliers in our restricted sample size. All error margins for observation means are reported as 1.96 standard errors for a 95% confidence interval.

## Results

### Study subjects

In total, 96 patients with Ewing sarcoma were included (age 19.9 ± 11.7; 41 women) into this study. The clinical characteristics of patients and properties of the final imaging datasets after curation and cleaning are depicted in Table 1. The majority of the Ewing sarcomas were located at the lower limb (thigh, lower leg and foot; 47.9% of all cases). The histological response was available for 60 of the 96 patients, as the remaining patients did not undergo surgery (Table S3 contains the list of patients with available histological response per MR imaging sequence). Out of these, 22 (36.67%) had a positive histological response.

**Table 1:** Patient characteristics, including: age, sex, treatment response measured as histological response, presence of metastasis at baseline and after the first cycle of chemotherapy (post-ct), and distribution of the general body locations in which Ewing sarcomas were found. * Data is given as mean ± standard deviation

Patient characteristics	
Age*	19.9 ± 11.7
**Sex**	
Men	56
Women	40
**Histological Response**	
< 10%	38
≥ 10%	22
N/A	36
**Metastasis**	
Baseline: Absent	69
Baseline: Present	26
Post-ct: Absent	86
Post-ct: Present	8
**Location**	
Thigh	26
Pelvis	22
Lower leg	17
Chest wall	9
Upper arm	9
Lower arm	6
Foot	3
Hand	2
Skull	1
Spine	1

### Determination of the best radiomics feature set for histological response detection

The detection of positive histological response was evaluated for all combinations of imaging modalities and scanning times, using all modeling strategies. Table 2 shows, for each defined time point, the performance of the best model compared to the clinical baseline model. Table S4 contains an extended view of the AUROC scores from all trained models for each time point and imaging sequence.

**Table 2:** Performance of the radiomics models that were statistically better than the clinical baseline model, as well as the optimal feature selection technique. * Data is given as mean ± 1.96 standard errors for a 95% confidence interval

Score	Relative delta T1fsgd	Baseline T2w	Post-ct T1fsgd	Clinical
	LogReg | Spearman	RFC | MRMR	RFC | MRMR	RFC
AUROC*	0.62 ± 0.03	0.61 ± 0.03	0.58 ± 0.02	0.53 ± 0.02
BA	0.57	0.60	0.57	0.53
F1	0.48	0.49	0.43	0.39
MCC	0.15	0.22	0.14	0.06
Sensitivity	0.47	0.48	0.42	0.40
Specificity	0.66	0.72	0.71	0.65

Three radiomics models performed better than the baseline model based on clinical features (AUROC of 0.53 ± 0.02). The relative delta of the T1fsgd imaging modality performed best of all assessed models with an AUROC of 0.62 ± 0.03 when trained with LogReg. The best baseline and post-ct models achieved respective AUROCs of 0.61 ± 0.03 and 0.58 ± 0.02, trained with RFCs. Interestingly, each of these models belongs to the different scan time points defined (baseline, post-ct, and absolute / relative delta) that is statistically better than the model based on clinical features.

**Fig. 3:**
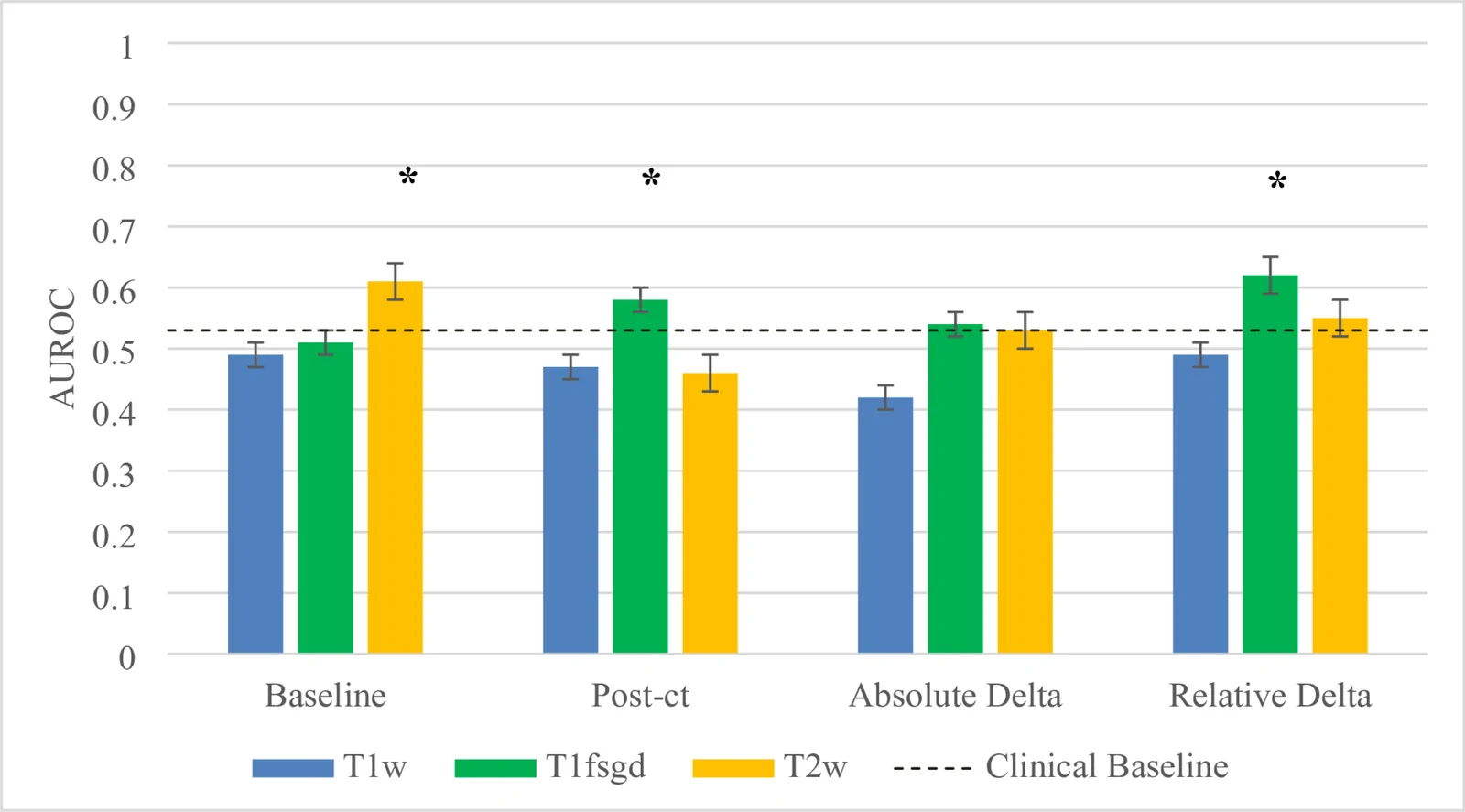
AUROC scores achieved by the best models for each combination of MRI modality (T1w, T1fsgd, T2w) and time point (at baseline, after the first cycle of chemotherapy (post-ct), absolute delta and relative delta time points). Clinical baseline represents the reference performance of the best model trained with clinical features. Asterisks are used to highlight models with statistical significance (p-value < 0.001) over the clinical baseline

None of the models utilizing T1w image features could outperform the clinical baseline models. The same is true for all the models utilizing T1fsgd features at baseline and T2w features at post-ct. An AUROC overview from best models per MR imaging sequence and time point can be found in Fig. 3. Furthermore, while we evaluated comprehensive feature fusion strategies (all baseline sequences, all relative delta-radiomics features, and the combination of radiomics features with clinical information or semantic features extracted from radiology readings clinical variables), these models yielded inferior predictive performance (Table S5).

### Comparison with Radiologists

Most discrete features from the radiological readings, including the encoded categorical features, exhibited minimal change between the baseline and post-ct time points. As a result, these semantic radiological features were largely not predictive. Of the continuous features, the maximum diameter of the extratumoral soft tissue component was the most informative predictor. The configuration for the best radiomics model was used to train models with the radiological readings features for each of the time points shown in Table 3. Additionally, Figure S2 shows an AUROC comparison of the best performing radiomics and radiological feature models.

**Table 3:** Performance of the semantic features obtained from radiological readings for the relative delta, baseline and post-ct time points. In all cases a LogReg was used with a two-step Spearman’s rank correlation coefficient as the feature selection technique. * Data is given as mean ± 1.96 standard errors for a 95% confidence interval

Score	Relative delta	Baseline	Post-ct
AUROC	0.58 ± 0.02	0.53 ± 0.02	0.49 ± 0.02
BA	0.53	0.52	0.51
F1	0.02	0.31	0.35
MCC	0.09	0.07	0.01
Sensitivity	0.15	0.03	0.42
Specificity	0.09	0.75	0.59

The point that provided the most insightful semantic features from radiological readings was the relative delta time point, yielding an AUROC of 0.58 ± 0.02 at a BA of 0.53 ± 0.02. Other clinically important metrics from this model performed insufficiently with an F1 score of 0.02, sensitivity of 0.15 and specificity of 0.09. The predictive performance of radiological models trained on features from the baseline and post-ct time points scored with an AUROC of 0.53 ± 0.02 and 0.49 ± 0.02, respectively.

### Feature importance

To gain more insight into the radiomics features contributing to the highest predictive performance, we conducted a feature importance analysis. Table 4 highlights a list of the 10 most predictive radiomics features for the best performing model. The top features shown have been selected based on the product of their average importance times their selection frequency.

**Table 4:** Feature importance report of the top 10 radiomics features from the best performing model (LogReg trained on the relative delta of T1fsgd features, selected with a two-step Spearman’s rank correlation coefficient). Score is calculated by multiplying the average importance of a feature by their selection frequency. Table S2 contains an extended description of the feature type names

Feature Type - Name	% Chosen	Importance	Score
Gldm - Small Dependence Low Gray Level Emphasis	80.7	0.11	0.09
Glrlm - Long Run Low Gray Level Emphasis	17.3	0.23	0.04
Glszm - Size Zone Non Uniformity	25.3	0.16	0.04
Glszm - Gray Level Non Uniformity Normalized	28.7	0.05	0.01
Glcm - Joint Entropy	16.7	0.08	0.01
Firstorder - Uniformity	8.7	0.12	0.01
Glcm - Difference Entropy	5.3	0.18	0.01
Shape - Minor Axis Length	14.7	0.06	0.01
Gldm - Gray Level Non Uniformity	10.0	0.07	0.01
Shape - Maximum D Diameter Row	9.3	0.06	0.01

Considering the 10 most important features for the best performing model, 7 of them were based on gray level values and their textural distribution. The most predictive feature was Small Dependence Low Gray Level Emphasis with an occurrence in 80.7% of the cases and an average importance of 0.11, for a final score of 0.09. This suggests that a high proportion of small, low gray-level pixel dependencies from non—enhanced regions, such as how fibrotic regions appear in the T1fsgd imaging, play a relevant role in the classification criteria of the model.

A patient-specific comparison of the 6 most predictive features between baseline and post-ct time points is illustrated in Figure S3. In all cases, features were significantly different between the baseline and post-ct distributions (Wilcoxon signed-rank test p-values < 0.05 [27]), showing a noticeable response to treatment in the histological response.

## Discussion

In this study, we analyzed the capabilities of MR imaging-based radiomics features compared to semantic features obtained from radiological readings to predict the outcome of neoadjuvant chemotherapy on Ewing sarcoma. Radiomics models from baseline T2w and relative delta T1fsgd achieved moderate discrimination of histologic viability, outperforming the models based on radiologically assessed morphological features.

The most important finding of our study is that baseline radiomics features from the T2w MR imaging sequence outperform the models based on semantic radiological features with an AUROC of 0.61 ± 0.03. The disappearance of such signal in post-treatment T2w features highlights the biological difficulty of assessing early chemotherapy response. At baseline, T2w imaging captures the native architecture, cellularity and water content of the untreated tumor. After chemotherapy treatment, the tumoral tissue undergoes different degrees of necrosis, inflammatory edema and early fibrosis. These non-specific tissue changes likely introduce significant biological heterogeneity that obscures the underlying signal of true histological viability, rendering post-treatment T2w radiomics less reliable for early prediction. To our knowledge, our analysis was based on the largest Ewing sarcoma dataset so far and emphasizes the trend of radiomics as a diagnostic staging tool that may be able to aid radiologists, tumor orthopedic surgeons and oncologists in therapeutic decisions [28]. Building on previous studies, which were either constrained by smaller datasets or focused solely on MRI-based radiomics, the evaluation carried out in our study incorporates semantic features obtained from expert radiological readings. The combination of loss in tumoral cell viability and tumor size provides a more comprehensive benchmark framework to support clinical decision-making.

The results of the relative delta of T1fsgd features, when considered within the radiomics analysis, appear reasonable, taking the effects of chemotherapy on vital tissue into consideration. In cases in which the neoadjuvant treatment is effective, the vital tumor tissue is reduced in volume, therefore the volume and amount of contrast agent uptake of the tumorous tissue declines as well. This decrease of the contrast enhanced tissue and signal intensity is a feature best portrayed in the relative delta of T1fsgd radiomics features. Altogether, the leading features cluster into multi-scale low-intensity emphasis, heterogeneity metric of non-uniformity and entropy, plus the lesion size and geometry. In the context of the T1fsgd imaging sequence, these indicate a region of hypo-intense signal at fine and coarse scales within a residual mass of variable elongation. The impact of changes in contrast enhancement on the prediction of the pathological response of STS to neoadjuvant treatment was also addressed in a previous study [29].

Considering contrast agent uptake on the gray level value scale, the impact of the change is also depicted within the feature importance. The two highest scoring features are both based on gray level values, albeit on low gray level values. This may correspond to the change of low grey value areas within a contrast-enhancing tumor in terms of tumor heterogeneity, which may reflect biological alterations such as a proportion change of tumoral and extratumoral tissue in the observed area.

This observation is in concordance with the morphological radiological features. Here, the main feature after pre-processing, redundancy reduction and filtering was the maximum diameter of the extratumoral soft tissue component. Other features did not show a significant change between baseline and post-ct to reflect any meaningful evolution in the relative delta, nor did they convey enough information to be kept after feature selection. Thus, the semantic radiological features were primarily reduced to the measurement of a tumor volume surrogate. This reduction highlights a fundamental challenge in comparing machine learning models with human clinical judgment: while radiologists utilize a comprehensive and highly nuanced assessment of the tumor environment, including subtle changes in necrosis patterns, edema, and lobulation, translating these qualitative interpretations into discrete semantic tags often yields high inter-rater variability that fails statistical inclusion thresholds. To more accurately and fairly benchmark radiomics against an expert assessment from radiologists, future studies should focus on extracting continuous semantic features, such as the volumetric percentage of necrosis, or implementing high-granularity semi-quantitative scoring systems. These continuous clinical variables are likely to be more mathematically stable and sensitive to longitudinal changes.

This study carries several limitations. First, due to the type of lesion and selection criteria, the sample size was small overall. Consequently, there is a distribution difference between those patients who have a measured histological response and those who did not due to the resectability criteria followed, which can involve the tumor location, stage, and treatment followed. Most likely, the selection may have reduced the maximum predictive performance which is represented by overall low predictive capabilities. Therefore, this level of performance is rather hypothesis-generating, than support a clinical decision, unless a complementary role is assumed. Second, due to the small size of the patient dataset, we decided to keep all patients within each subgroup to achieve patient groups as large as possible. Still, this may not be enough for the model to fully capture all patterns within the data, and a larger cohort may be needed. Restricting the patient cohort to patients that would have had all imaging scans available would have largely undermined model development. Furthermore, the limited sample size also constrained our methodological approach. While advanced non-linear machine learning architectures and more complex synthetic data augmentation can theoretically capture highly complex feature interactions, they are heavily prone to overfitting on small clinical datasets while not being directly clinically interpretable. Consequently, we restricted our pipeline to simpler models to prioritize generalizability and robustness. Thus, model evaluation should be made as an exploratory approach. To counteract this problem, we performed repeated nested cross validation to obtain maximum robust statistical measures of performance.

Finally, our study focused mainly on structural MR imaging sequences. We see potential for future research assessing the predictive capabilities of diffusion-weighted imaging (DWI) and dynamic contrast-enhanced (DCE) MRI. The biological insights that these sequences provide, especially the tumor cellularity and diffusion properties from DWI, would seamlessly align with the histological response assessment used in this research work. Given protocol era and data availability, potential future research should address a multimodal approach encompassing these findings with other traditional imaging methods and measurements. In addition, leveraging more highly parameterized architectures and multi-sequence feature fusion strategies remains a critical path for future research, provided that larger datasets can be aggregated through wider Ewing sarcoma consortia.

## Conclusion

In this study, we evaluated the question of whether radiomics can predict histologic viability as a complementary signal for Ewing sarcoma response to neoadjuvant chemotherapy from longitudinal MR imaging data, and we compared this with the assessment of morphological radiological features. The MR imaging radiomics based prediction utilizing baseline radiomics and delta radiomics (difference between post-ct features and baseline features, then divided by baseline features) of the response to neoadjuvant chemotherapy of Ewing sarcoma outperformed the models based on morphological, radiologically assessed features. Consequently, radiomics baseline and post-ct MR imaging data may potentially be a useful supplementary tool for the early prediction of the response to neoadjuvant treatment together with volume assessment or in a posterior multimodal scenario and, therefore, for the prognosis of patients with Ewing sarcoma.

## Supplementary information

Below is the link to the electronic supplementary material.

Supplementary Material 1

## Data Availability

All data and code used in this research is available upon contact of the correspondence author (Óscar Llorián-Salvador, oscar.llorian-salvador@tum.de) and in concordance to the ethics committee of the TUM University hospital.

## Abbreviations

AUROC: area under the receiver operating characteristic curve
BA: balanced accuracy
CI: confidence interval
CT: computed tomography
DCE: dynamic contrast-enhanced
DICOM: Digital Imaging and Communications in Medicine
DWI: diffusion-weighted imaging
F1: F1 score
GD: gadolinium
GLCM: gray-level co-occurrence matrix
GLDM: gray-level dependence matrix
GLRLM: gray-level run-length matrix
GLSZM: gray-level size-zone matrix
IBSI: Image Biomarker Standardization Initiative
ICC: intraclass correlation coefficient
IRB: institutional review board
LASSO: least absolute shrinkage and selection operator
LogReg: logistic regression
MCC: Matthews correlation coefficient
ML: machine learning
MRI: magnetic resonance imaging
MRMR: minimum redundancy-maximum relevance
NIfTI: Neuroimaging Informatics Technology Initiative
PCA: principal component analysis
PET: positron emission tomography
PET/CT: positron emission tomography/computed tomography
RECIST: Response Evaluation Criteria in Solid Tumors
RFC: random forest classifier
SMOTE: synthetic minority oversampling technique
STS: soft tissue sarcoma
SVM: support vector machine
T1fsgd: T1-weighted fat-saturated imaging with contrast agent
T1w: T1-weighted imaging
T2w: T2-weighted imaging
VOI: volume of interest
