# Supplementary material for: Can radiomics outperform radiologists in predicting Ewing sarcoma response to neoadjuvant chemotherapy from longitudinal MRI?
Source: BMC Cancer. 2026 Nov 12;26:1407. doi: 10.1186/s12885-026-17332-0 (PMC13761021; doi:10.1186/s12885-026-17332-0)
Supplement: Supplementary file 1 [file 12885_2026_17332_MOESM1_ESM.docx]

**Supplemental Material**

[Supplemental Tables	2](#_Toc227826635)

[TABLE S1	2](#_Toc227826636)

[TABLE S2	2](#_Toc227826637)

[TABLE S3	3](#_Toc227826638)

[TABLE S4	3](#_Toc227826639)

[TABLE S5	4](#_Toc227826640)

[TABLE S6	4](#_Toc227826641)

[TABLE S7	5](#_Toc227826642)

[Supplemental Figures	6](#_Toc227826643)

[FIGURE S1	6](#_Toc227826644)

[FIGURE S2	6](#_Toc227826645)

[FIGURE S3	7](#_Toc227826646)

[REFERENCES	9](#_Toc227826647)

# Supplemental Tables

## TABLE S1

**MRI acquisition parameters**

| **Acquisition Parameters** | Magnetic Field Strength (T) | | | Repetition Time (ms) * | Echo Time (ms) * | Slice Thickness (mm) * | Flip Angle (°) * | Pixel Spacing (mm) * |
| --- | --- | --- | --- | --- | --- | --- | --- | --- |
|  | 1.0 | 1.5 | 3.0 |  |  |  |  |  |
| **Baseline** | 3 | 63 | 30 | 1559 - 2603.2 | 23.9 - 40.1 | 4.4 - 5.2 | 101.8 - 232.2 | 0.66 - 0.82 x 0.66 - 0.82 |
| **Follow Up** | 6 | 55 | 34 | 1875.5 - 2959.3 | 26.8 - 39.6 | 4.7 - 5.3 | 111.6 - 131.8 | 0.75 - 0.91 x 0.75 - 0.91 |

Table S1. Acquisition parameters extracted from the DICOM metadata files. * Data is given as the 95% confidence interval boundary values.

## TABLE S2

**Extracted radiomics features**

| **Shape Features** | |
| --- | --- |
| Volume | Surface Area |
| Surface Volume Area | Sphericity |
| Spherical Disproportion | Maximum 3D Diameter |
| Maximum 2D Diameter Slice | Maximum 2D Diameter Column |
| Maximum 2D Diameter Row | Major Axis |
| Minor Axis | Least Axis |
| Elongation | Flatness |
| **First Order Features** | |
| Energy | Intensity Histogram Entropy |
| Minimum | 10th Percentile |
| 90th Percentile | Maximum |
| Mean | Median |
| Interquartile Range | Range |
| Mean Absolute Deviation (MAD) | Robust Mean Absolute Deviation (rMAD) |
| Root Mean Squared (RMS) | Skewness |
| Excess Kurtosis | Variance |
| Intensity Histogram Uniformity |  |
| **Gray Level Co-occurrence Matrix (GLCM) Features** | |
| Autocorrelation | Joint Average |
| Cluster Prominence | Cluster Shade |
| Cluster Tendency | Contrast |
| Correlation | Difference Average |
| Difference Entropy | Difference Variance |
| Joint Energy (IBSI: Angular Second Moment) | Joint Entropy |
| Informal Measure of Correlation (IMC) 1 | Informal Measure of Correlation (IMC) 2 |
| Inverse Difference Moment (IDM) | Inverse Difference Moment Normalized (IDMN) |
| Inverse Difference (ID) | Inverse Difference Normalized (IDN) |
| Inverse Variance | Maximum Probability (IBSI: Joint maximum) |
| Sum Entropy | Sum of Squares (IBSI: Sum of Squares) |
| Maximal Correlation Coefficient (MCC) |  |
| **Gray Level Size Zone Matrix (GLSZM) Features** | |
| Small Area Emphasis (SAE) | Large Area Emphasis (LAE) |
| Gray Level Non-Uniformity (GLN) | Gray Level Non-Uniformity Normalized (GLNN) |
| Size-Zone Non-Uniformity (SZN) | Size-Zone Non-Uniformity Normalized (SZNN) |
| Zone Percentage (ZP) | Gray Level Variance (GLV) |
| Zone Variance (ZV) | Zone Entropy (ZE) |
| Low Gray Level Zone Emphasis (LGLZE) | High Gray Level Zone Emphasis (HGLZE) |
| Small Area Low Gray Level Emphasis (SALGLE) | Small Area High Gray Level Emphasis (SAHGLE) |
| Large Area Low Gray Level Emphasis (LALGLE) | Large Area High Gray Level Emphasis (LAHGLE) |
| **Gray Level Run Length Matrix (GLRLM) Features** | |
| Short Run Emphasis (SRE) | Long Run Emphasis (LRE) |
| Gray Level Non-Uniformity (GLN) | Gray Level Non-Uniformity Normalized (GLNN) |
| Run Length Non-Uniformity (RLN) | Run Length Non-Uniformity Normalized (RLNN) |
| Run Percentage (RP) | Gray Level Variance (GLV) |
| Run Variance (RV) | Run Entropy (RE) |
| Low Gray Level Run Emphasis (LGLRE) | High Gray Level Run Emphasis (HGLRE) |
| Short Run Low Gray Level Emphasis (SRLGLE) | Short Run High Gray Level Emphasis (SRHGLE) |
| Long Run Low Gray Level Emphasis (LRLGLE) | Long Run High Gray Level Emphasis (LRHGLE) |
| **Neighbouring Gray Tone Difference Matrix (NGTDM) Features** | |
| Coarseness | Contrast |
| Busyness | Complexity |
| Strength |  |
| **Gray Level Dependence Matrix (GLDM) Features** | |
| Small Dependence Emphasis (SDE) | Large Dependence Emphasis (LDE) |
| Gray Level Non-Uniformity (GLN) | Dependence Non-Uniformity (DN) |
| Dependence Non-Uniformity Normalized (DNN) | Gray Level Variance (GLV) |
| Dependence Variance (DV) | Dependence Entropy (DE) |
| Low Gray Level Emphasis (LGLE) | High Gray Level Emphasis (HGLE) |
| Small Dependence Low Gray Level Emphasis (SDLGLE) | Small Dependence High Gray Level Emphasis (SDHGLE) |
| Large Dependence Low Gray Level Emphasis (LDLGLE) | Large Dependence High Gray Level Emphasis (LDHGLE) |

Table S2. Radiomics features extracted from the MRI scans. All extracted features were computed according to the “image biomarker standardization initiative” (IBSI) guidelines [1]. The pyRadiomics package (version 2.0) implemented in Python (version 3.6.4) was used for feature extraction [2].

## TABLE S3

**MRI modality availability**

| Imaging modality | Baseline | Post-ct |
| --- | --- | --- |
| T1w | 81 (55) | 74 (49) |
| T1fsgd | 73 (46) | 81 (53) |
| T2w | 58 (37) | 66 (42) |

Table S3. Number of available MR imaging sequences, with the subset in which the histological response was available after surgical resection in parenthesis.

## TABLE S4

**AUROC performance of all radiomics models**

| **AUROC** | LogReg | LASSO | SVM | RFC |
| --- | --- | --- | --- | --- |
| Baseline T1w | 0.49 ± 0.02 | 0.49 ± 0.02 | 0.48 ± 0.02 | 0.47 ± 0.02 |
| Post-ct T1w | 0.45 ± 0.02 | 0.45 ± 0.02 | 0.47 ± 0.02 | 0.42 ± 0.02 |
| Absolute delta T1w | 0.39 ± 0.02 | 0.42 ± 0.02 | 0.42 ± 0.02 | 0.38 ± 0.02 |
| Relative delta T1w | 0.47 ± 0.02 | 0.47 ± 0.02 | 0.49 ± 0.02 | 0.43 ± 0.02 |
| Baseline T1fsgd | 0.46 ± 0.02 | 0.48 ± 0.02 | 0.48 ± 0.02 | 0.51 ± 0.02 |
| Post-ct T1fsgd | 0.56 ± 0.02 | 0.55 ± 0.02 | 0.54 ± 0.02 | 0.58 ± 0.02 |
| Absolute delta T1fsgd | 0.51 ± 0.02 | 0.49 ± 0.02 | 0.51 ± 0.02 | 0.54 ± 0.02 |
| Relative delta T1fsgd | 0.62 ± 0.03 | 0.62 ± 0.03 | 0.59 ± 0.03 | 0.50 ± 0.03 |
| Baseline T2w | 0.56 ± 0.02 | 0.55 ± 0.02 | 0.54 ± 0.03 | 0.61 ± 0.03 |
| Post-ct T2w | 0.43 ± 0.02 | 0.42 ± 0.02 | 0.46 ± 0.02 | 0.44 ± 0.03 |
| Absolute delta T2w | 0.52 ± 0.03 | 0.53 ± 0.03 | 0.52 ± 0.03 | 0.53 ± 0.03 |
| Relative delta T2w | 0.49 ± 0.03 | 0.50 ± 0.03 | 0.47 ± 0.03 | 0.55 ± 0.03 |
| Clinical | 0.41 ± 0.02 | 0.42 ± 0.02 | 0.51 ± 0.02 | 0.53 ± 0.02 |

Table S4. AUROC scores achieved by all radiomics models, which include all considered MRI modalities (T1w, T1fsgd, T2w); all defined time points (baseline, post-ct, absolute delta and relative delta); and for LogReg, LASSO, SVM and RFC. Values are given as mean ± 1.96 standard errors for a 95% confidence interval.

## TABLE S5

**Model performance of combined datasets**

| **Data Combination** | **Modeling strategy** | **AUROC** |
| --- | --- | --- |
| Relative delta T1fsgd | *LogReg \| Spearman* | 0.62 ± 0.03 |
| Relative delta T1fsgd & Clinical | *LogReg \| MRMR* | 0.55 ± 0.03 |
| Radiology readings & Clinical | *LogReg \| MRMR* | 0.52 ± 0.04 |
| Relative delta T1fsgd & Radiology readings | *LogReg \| MRMR* | 0.46 ± 0.06 |
| Combined Baselines Radiomics | *RF \| MRMR* | 0.52 ± 0.03 |
| Combined Delta Radiomics | *LASSO \| MRMR* | 0.41 ± 0.03 |

Table S5. Predictive performance of different combinations of modality and MRI sequences. * Data is given as mean ± 1.96 standard errors for a 95% confidence interval

## TABLE S6

**Baseline distribution of manufacturers and magnetic field strengths**

| **Manufacturer Model Name** | **Magnetic Field Strength / Tesla** | | |
| --- | --- | --- | --- |
|  | **1** | **1.5** | **3** |
| Achieva | 0 | 7 | 9 |
| Achieva dStream | 0 | 2 | 2 |
| Aera | 0 | 7 | 0 |
| Avanto | 0 | 21 | 0 |
| Biograph_mMR | 0 | 0 | 1 |
| DISCOVERY MR750 | 0 | 0 | 1 |
| Espree | 0 | 2 | 0 |
| Harmony | 1 | 0 | 0 |
| HarmonyExpert | 1 | 0 | 0 |
| Ingenia | 0 | 0 | 9 |
| Intera | 0 | 4 | 0 |
| Intera Achieva | 0 | 1 | 0 |
| MAGNETOM Amira | 0 | 1 | 0 |
| MAGNETOM EXPERT | 1 | 0 | 0 |
| MAGNETOM VISION | 0 | 1 | 0 |
| MAGNETOM_ESSENZA | 0 | 1 | 0 |
| OPTIMA MR360 | 0 | 2 | 0 |
| SIGNA EXCITE | 0 | 1 | 0 |
| Signa HDxt | 0 | 2 | 0 |
| Skyra | 0 | 0 | 1 |
| Sonata | 0 | 1 | 0 |
| Spectra | 0 | 0 | 1 |
| Symphony | 0 | 7 | 0 |
| SymphonyTim | 0 | 1 | 0 |
| Verio | 0 | 0 | 6 |
| N/A | 0 | 2 | 0 |

Table S6. Distribution of Manufacturer model names for the three possible values of magnetic field strengths (1, 1.5 and 3 Tesla) at baseline time. Chi-square test for the association between both variables p-value = 9.74e-15

## TABLE S7

**Post-ct distribution of manufacturers and magnetic field strengths**

| **Manufacturer Model Name** | **Magnetic Field Strength / Tesla** | | |
| --- | --- | --- | --- |
|  | **1** | **1.5** | **3** |
| Achieva | 0 | 5 | 4 |
| Achieva dStream | 0 | 0 | 1 |
| Aera | 0 | 12 | 0 |
| Avanto | 0 | 25 | 0 |
| Avanto_fit | 0 | 1 | 0 |
| Biograph_mMR | 0 | 0 | 3 |
| Harmony | 4 | 0 | 0 |
| Ingenia | 0 | 0 | 14 |
| Ingenia S | 0 | 1 | 0 |
| Intera | 1 | 1 | 0 |
| Intera Achieva | 0 | 1 | 0 |
| MAGNETOM EXPERT | 1 | 0 | 0 |
| MAGNETOM VISION | 0 | 4 | 0 |
| Skyra | 0 | 0 | 3 |
| Symphony | 0 | 4 | 0 |
| SymphonyTim | 0 | 1 | 0 |
| Verio | 0 | 0 | 9 |
| N/A | 0 | 1 | 0 |

Table S7. Distribution of Manufacturer model names for the three possible values of magnetic field strengths (1, 1.5 and 3 Tesla) after the first cycle of chemotherapy. Chi-square test for the association between both variables p-value = 6.06e-21

# Supplemental Figures

## FIGURE S1

**Patient workflow**

Figure S1. Patient cohort and dataset creation. Histological samples taken after the first chemotherapy cycle were evaluated according to the Salzer-Kuntschik score for histological response [3]. For appropriate imaging, MR imaging scans before (baseline) and after (post-ct) were mandatory. Due to technical issues during the radiomics analysis, two more patients had to be excluded.

## FIGURE S2

**AUROC score comparison of the best radiomics and radiology models**

AUROC

|  | Radiology | Radiomics |
| --- | --- | --- |
| Baseline | 0.53 | 0.61 |
| Post-ct | 0.49 | 0.58 |
| Relative Delta | 0.58 | 0.62 |

|  | Clinical Baseline |
| --- | --- |
| 1 | 0.53 |
| 2 | 0.53 |
| 3 | 0.53 |
| 4 | 0.53 |
| 5 | 0.53 |
| 6 | 0.53 |
| 7 | 0.53 |
| 8 | 0.53 |
| 9 | 0.53 |
| 10 | 0.53 |
| 11 | 0.53 |
| 12 | 0.53 |
| 13 | 0.53 |
| 14 | 0.53 |
| 15 | 0.53 |
| 16 | 0.53 |
| 17 | 0.53 |
| 18 | 0.53 |
| 19 | 0.53 |
| 20 | 0.53 |
| 21 | 0.53 |
| 22 | 0.53 |
| 23 | 0.53 |
| 24 | 0.53 |

Figure S2. AUROC values of baseline, post-ct and relative delta time points of the best performing radiology (blue) and radiomics (green; T1fsgd MRI modality) models.

## FIGURE S3

**Patient-wise comparison of the 6 most predictive features between baseline and post-ct**

Figure S3. Evolution from baseline to post-ct of all patients for the 6 most predictive features in the best performing model: a LogReg trained on the relative delta of T1fsgd features selected by a two-step Spearman’s correlation coefficient. Each radiomics feature is accompanied by the p-value of the Wilcoxon signed-rank test.

# REFERENCES

1.	Zwanenburg, A.; Vallières, M.; Abdalah, M.A.; Aerts, H.J.W.L.; Andrearczyk, V.; Apte, A.; Ashrafinia, S.; Bakas, S.; Beukinga, R.J.; Boellaard, R.; et al. The Image Biomarker Standardization Initiative: Standardized	Quantitative Radiomics for High-Throughput Image-Based	Phenotyping. *Radiology* **2020**, *295*, 328–338, doi:10.1148/radiol.2020191145.

2.	van Griethuysen, J.J.M.; Fedorov, A.; Parmar, C.; Hosny, A.; Aucoin, N.; Narayan, V.; Beets-Tan, R.G.H.; Fillion-Robin, J.-C.; Pieper, S.; Aerts, H.J.W.L. Computational Radiomics System to Decode the Radiographic Phenotype. *Cancer Research* **2017**, *77*, e104–e107, doi:10.1158/0008-5472.CAN-17-0339.

3.	Salzer-Kuntschik, M.; Brand, G.; Delling, G. Determination of the Degree of Morphological Regression Following Chemotherapy in Malignant Bone Tumors. *Der Pathologe* **1983**, *4*, 135–141.
